# Host plant nutrient contents influence nutrient contents in *Bradysia cellarum* and *Bradysia impatiens*

**DOI:** 10.1371/journal.pone.0226471

**Published:** 2020-04-29

**Authors:** Yuping Gou, Peter Quandahor, Yanxia Zhang, Jeffrey A. Coulter, Changzhong Liu

**Affiliations:** 1 College of Plant Protection, Gansu Agricultural University, Anning District, Lanzhou, Gansu Province, P. R. China; 2 Biocontrol Engineering Laboratory of Crop Diseases and Pests of Gansu Province, Anning District, Lanzhou, Gansu Province, P. R. China; 3 Department of Agronomy and Plant Genetics, University of Minnesota, St. Paul, Minnesota, United States of America; Chinese Academy of Agricultural Sciences Institute of Plant Protection, CHINA

## Abstract

The chive maggot *Bradysia cellarum* and the fungus gnat *B*. *impatiens* are two primary root pests of plants, which can coexist on the same host plants and are the devastating pests on liliaceous crops and edible fungi. Their growth and development are affected by the nutrient contents of their host plants. In this study, we assessed the effects of different host plant nutrients on the nutrient contents of these two *Bradysia* species. The nutrients of the chive (*Allium tuberosum* Rottl. ex Spreng.), board bean (*Vicia faba* L.), lettuce (*Lactuca sativa* L. var. ramosa Hort.), cabbage (*Brassica oleracea* L.), wild cabbage (*Brassica oleracea* var. capitata rubra) and pepper (*Capsicum annuum* L.) roots were determined, and their effect on nutrient content of the two *Bradysia* species after feeding on the host plant for three continuous generations were evaluated. The results show that chive and B-bean contained higher levels of protein, free amino acid, soluble sugar and starch than others. As a result, the soluble sugar, fat and protein contents were significantly higher in both *Bradysia* species reared on chive and B-bean than on cabbage, lettuce, W-cabbage and pepper, suggesting nutritional preference of these insects. Based on our results, we concluded that the two *Bradysia* species displayed nutrient preference toward chive and B-bean, which provides a reference for understanding their host plant range and for control of the insect species via field crop rotations.

## Introduction

The chive maggot *Bradysia cellarum* (Frey, 1948) (= *Bradysia odoriphaga* Yang and Zhang, 1985) and the fungus gnat *Bradysia impatiens* (Johnnsen, 1912) (= *Bradysia difformis* Frey, 1948), are two main root insect pests that belong to the family Diptera and the genus Sciaridae [1, 2]. They may coexist on the same host plants and devastate liliaceous crops and edible fungi [3]. The larvae of these species attack the host plants by chewing or stripping plant roots, especially the young, and developing root hairs and stems of seedlings, leading to losses in crop production and hindering agricultural productivity as well as farmers’ income [2, 4]. In the fields, the two *Bradysia* species occur with similar regularities that they outbreak in spring and autumn, while populations decline in summer [4]. The chive maggot *B*. *cellarum* heavily attacks chive, onion, garlic, cabbage, and watermelon seedlings [5–8]. The fungus gnat *B*. *impatiens* [9] also causes damage to chive, lily, green onion, garlic, B-bean, cabbage, butterfly orchid, and jonquil [10–13], which was first recorded on *Pleurotus ostreatus* and *Agrocybe chaxingu* in Yunnan, China in 2009 [14, 15].

Growth, development, and reproduction of plant-feeding insects depend on finding suitable host plants, which provide favorite nutrition for them. It is found that there are significant differences among different host plants for the developmental duration, life longevity, and population trend index of the fruit fly (*Bactrocera*), indicating that the fly’s population is influenced by host plant nutrition [16]. Protein, amino acid, soluble sugar and starch are the main nutrients in plants, which have a significant influence on the growth, reproduction, survival rate and spawning of herbivores [17]. For example, high soluble sugar content in wheat plants caused a high intrinsic rate of increase, short development time of the nymph, and fecundity in the aphid species *Rhopalosiphum padi* L. [18]. The soluble sugar content in the plant was negatively correlated with aphid resistance but positively correlated with fecundity [19, 20]. It was reported that the total carbohydrate content of different plants had no significant effect on the growth and development of the beet armyworm *Spodoptera exigua* (Hubner), but positively affected its oviposition and larval stage development [21]. Starch is a type of polysaccharide in plants and plays an important role in storing energy, which has effect on the feeding, growth and reproduction of phytophagous insects, for instance, *Helicoverpa armigera* [22]. The nutrient content of host plants may also be one of the important factors that determines host plant selection for insects [22].

Adults of *B*. *cellarum* and *B*. *impatiens* do not feed, so their primary nutrients are accumulated and come from larval stages. Research on the biology [23], prevention and treatment [24], morphology [6] and sex pheromones [25] of *B*. *cellarum* and *B*. *impatiens* have attracted much attention in recent years. However, the effects of host plant nutrients on the nutrient contents of these two *Bradysia* species have not been reported. Since nutrients are varied, which could affect development of herbivorous insects. Understanding the relation of the host plant nutrient contents and the insect feeding and growth is important for preventing and controlling insect pests of crops.

Several studies have reported on the conversion of host plants food nutrients into insect’s performance, such as growth, development, survival, reproduction and other important functions [16–21]. Primary nutrients in insects functions as the qualitative and quantitative chemical substances essential for molting process, spermatogenesis (the manufacture of sperms), oogenesis (the manufacture of eggs), synthesis of pigment and reproduction, detoxification process, and resistance mechanism [26]. Moreover, nutrients stored within the insect egg are reported to support limited development and, in case of primary nutrients, supply sufficient quantity to ensure development of one generation. Lack of essential amino acid is also reported to affect larval development [26].

In this study, six host plants of *B*. *cellarum* and *B*. *impatiens*, including chive, B-bean (board bean), lettuce, cabbage, W-cabbage (wild cabbage), and pepper were selected for determination of protein, free amino acid, soluble sugar and starch contents in the roots. Correspondingly, the soluble sugar, glycogen, total fat, neutral fat and protein in two *Bradysia* species were examined after feeding on those host plants for more than three continuous generations. Furthermore, the potential effects of host plant nutrients on the nutrient content of the two *Bradysia* species were evaluated. The results demonstrated that the two *Bradysia* species displayed nutrient preference toward chive and B-bean, which provides a reference for understanding their host plant range and for control of the insect species via field crop rotations.

## Materials and methods

### Host plants

Chive (*Allium tuberosum*), B-bean (*Vicia faba*), lettuce (*Lachca sativa*), cabbage (*Brassica pekinensis*), W-cabbage (*Brassica oleracea*), and pepper (*Capsicum annuum*) were seeded in the laboratory of Gansu Agricultural University (36°5’20” N, 103°41’54” E), Lanzhou, China. Each species were planted in three pots. Two months after seeding, the stems of each host plant species were collected to feed the larvae of the two *Bradysia* species. Plant roots were extracted and cleaned with water. Their nutrient contents were then determined individually.

### Insects collection and treatment

Populations of *B*. *cellarum* and *B*. *impatiens* were initially collected from chive fields in Gangu county (34°44’44” N, 105°20’13” E), Gansu Province, China. It was reared with stems of each tested host plant, where eggs, larvae and pupae were mass cultured in moisturized petri dishes (12 cm) containing filter papers (1 upper cover and 2 lower covers), and the fresh diets were replaced daily [27–29]. Once the adults emerged, they were transferred into plastic containers (15 × 9cm) with moist filter papers. Third instar larvae from colony for more than three continuous generations were used for the experiments. Four larvae of two *Bradysia* insect reared on each diets were collected in a 2 mL centrifuge tube and stored in a refrigerator at -80 °C for determination of nutrients with three replicates.

### Determination of nutrients in tested plants

The roots from three pots of the six host plants were used to determine the contents of soluble protein, free amino acid, soluble sugar and starch separately. Each experiment was repeated three times. Protein content was measured using the Bradford assay method [30]. Optical density (OD) values were measured at 595 nm, and protein content was calculated based on the standard curve of BSA (Sangon biotech, Shanghai, China). Free amino acid content was detected by the ninhydrin method [30]. The OD value was measured at 580 nm and calculated based on the standard curve of leucine. Soluble sugar content was determined using the anthrone colorimetry method and calculated based on the standard curve of D-glucose (Sangon biotech, Shanghai, China) [30].

### Carbohydrate determination in the insect body

Soluble sugar and the glycogen contents of the two *Bradysia* species reared with different host plants were determined using the methods of as described previously [30]. Briefly, 160 μL of the homogenate suspension was transferred into a 2 mL centrifuge tube, and mixed with 20 μL of 20% sodium sulphate and 1500 μL of a chloroform/methanol solution (1:2 v/v). The mixture was centrifuged at 10,000 rpm for 15 min at 4 °C. Then, the upper solution was transferred into a new centrifuge tube as a premeasured solution.

For determination of the soluble sugars of *Bradysia* larvae, 150 μL premeasured solution was transferred into a 1.5 mL centrifuge tube and then mixed with 10 μL ddH_2_O and 240 μL anthrone (1.42 g/L). The mixture was incubated at 25 °C for 10 min and then incubated in boiling water for another 10 min, followed by cooling to room temperature. The mixture was then transferred into a 96-pore coated plate used for determined of OD value at a 620 nm wavelength. The soluble sugar content was calculated based on the standard curve of D-glucose (Sangon biotech, Shanghai, China).

To determine the glycogen of *Bradysia* larvae, similarly, the premeasured solution was transferred into a new centrifuge tube and mixed with 400 μL of 80% methanol, which was homogenized in an ultrasonic cleaning apparatus for 5–10 min. After centrifugation at 10,000 rpm for 10 min at 4 °C. Then, the upper solution was mixed with anthrone solution, incubated for 10 min under room temperature, and then in boiling water for another 10 min. The mixture was then transferred into a 96-pore coated plate used for assay of OD at 620 nm. The glycogen content was calculated based on the standard curve of D-glucose.

### Total fat and neutral fat determination in the insect body

Total fat and neutral fat of *Bradysia* larvae were determined using the methods of as described previously [31]. To determine total fat content, 100 μL of the premeasured solution was transferred to a 1 mL centrifuge tube, then dried at 90 °C until all solvents were completely evaporated. Then, 10 μL of 98% sulphuric acid and 190 μL of vanillin solution (1.2 g/L) were added and incubated at room temperature for 15 min. The mixture was transferred into a 96-pore coated plate used for determination of OD value at 525 nm. The total fat content was calculated based on the standard curve of triolein (Sigma, St. Louis, MO, USA).

For detection of neutral fat, 150 μL of the premeasured solution was transferred into a 1.5 mL centrifuge tube and dried at 90 °C until all solvents were completely evaporated. One mL of chloroform was added to dissolve the pellet and centrifuged at 10,000 rpm for 15 min at 4 °C. After centrifugation, 100 μL of the upper solution was treated as same as the procedure examining the total fat. The OD value of neutral fat was measured at 525 nm, and its content was calculated based on the standard curve of triethylhexanoin (Aladdin, Shanghai, China).

### Protein determination in insect body

Protein content of the two *Bradysia* species in the different treatments was determined using a method as described previously [31]. Briefly, 20 μL of the homogenate suspension was transferred into a 96-well plate and then mixed with 200 μL of Coomassie brilliant blue G-250 dye (Bradford assay) for 15–20 min. The OD values were measured at 595 nm, and protein content was calculated based on the standard curve of BSA (Sangon biotech, Shanghai, China).

### Statistical analysis

The Proc Means program (SPSS 19.0, IBM, Armonk, NY, USA) was used for one-way analysis of variance [32], Tukey’ S HSD was used to assess differences among means [33], and bivariate correlation was used to calculate the correlation coefficients.

## Results

### Nutrient contents in roots of different host plants

**Fig 1:**
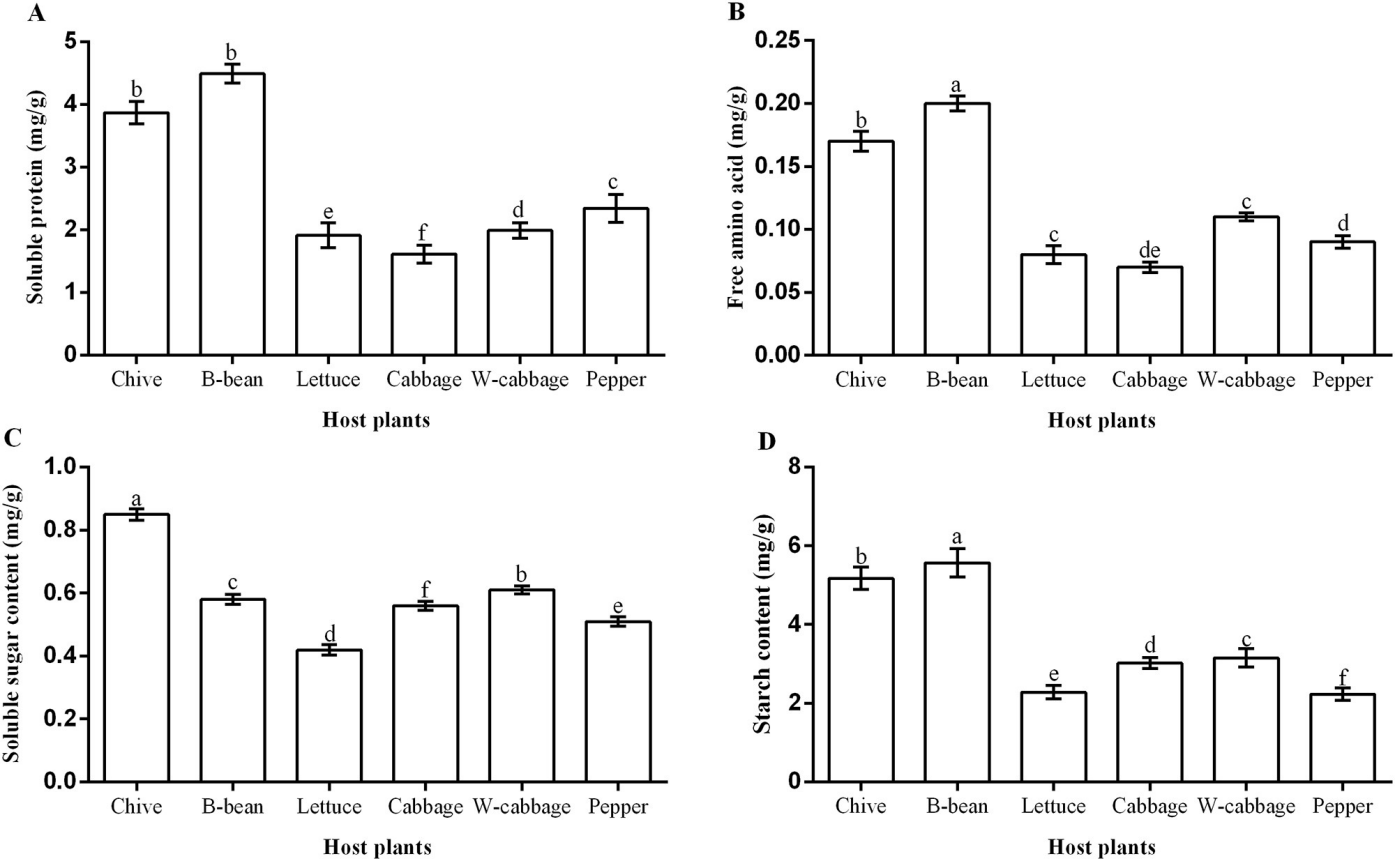
Nutrient contents in roots of the host plant. A: Protein content; B: Free amino acid content; C: Soluble sugar content; D: Starch content. Different small letters indicate a significant difference at the 0.05 level among treatments of different host plants (Tukey’ s HSD).

Protein content in roots of the six tested host plants is shown in Fig 1A. The highest content was detected in B-bean roots (4.49 mg/g), followed by chive (3.87 mg/g), pepper (2.34 mg/g), W-cabbage (1.99 mg/g), lettuce (1.91 mg/g), and cabbage (1.61 mg/g). No statistical difference was detected in protein content between B-bean and chive. However, the contents in these two plants were significantly (*P < 0*.*05*) higher than other host plants (Fig 1A).

Free amino acid contents were 0.20 mg/g for B-bean, 0.17 mg/g for chive, 0.11 mg/g for W-cabbage, 0.99 mg/g for pepper, 0.08 mg/g, and 0.07 mg/g in turn from the high levels to low levels. (Fig 1B). Roots soluble sugar content in the six tested host plants was 0.85 mg/g in chive, followed by 0.61 mg/g in W-cabbage, 0.58 mg/g in B-bean, 0.56 mg/g in cabbage, 0.51 mg/g in pepper, and 0.42 mg/g in lettuce (Fig 1C). The highest starch content among the tested host plants occurred in the roots of B-bean (5.57 mg/g), while the lowest starch content was detected in the roots of pepper (2.23 mg/g) (Fig 1D). Root starch content of other host plants was 5.18 mg/g for chive, 3.15 mg/g for W-cabbage, 3.02 mg/g for cabbage, and 2.28 mg/g for lettuce. Starch content was significantly (*P < 0*.*05*) higher in the roots of B-bean and chive than other host plants.

### Soluble sugar content in *B*. *cellarum* and *B*. *impatiens*

**Fig 2:**
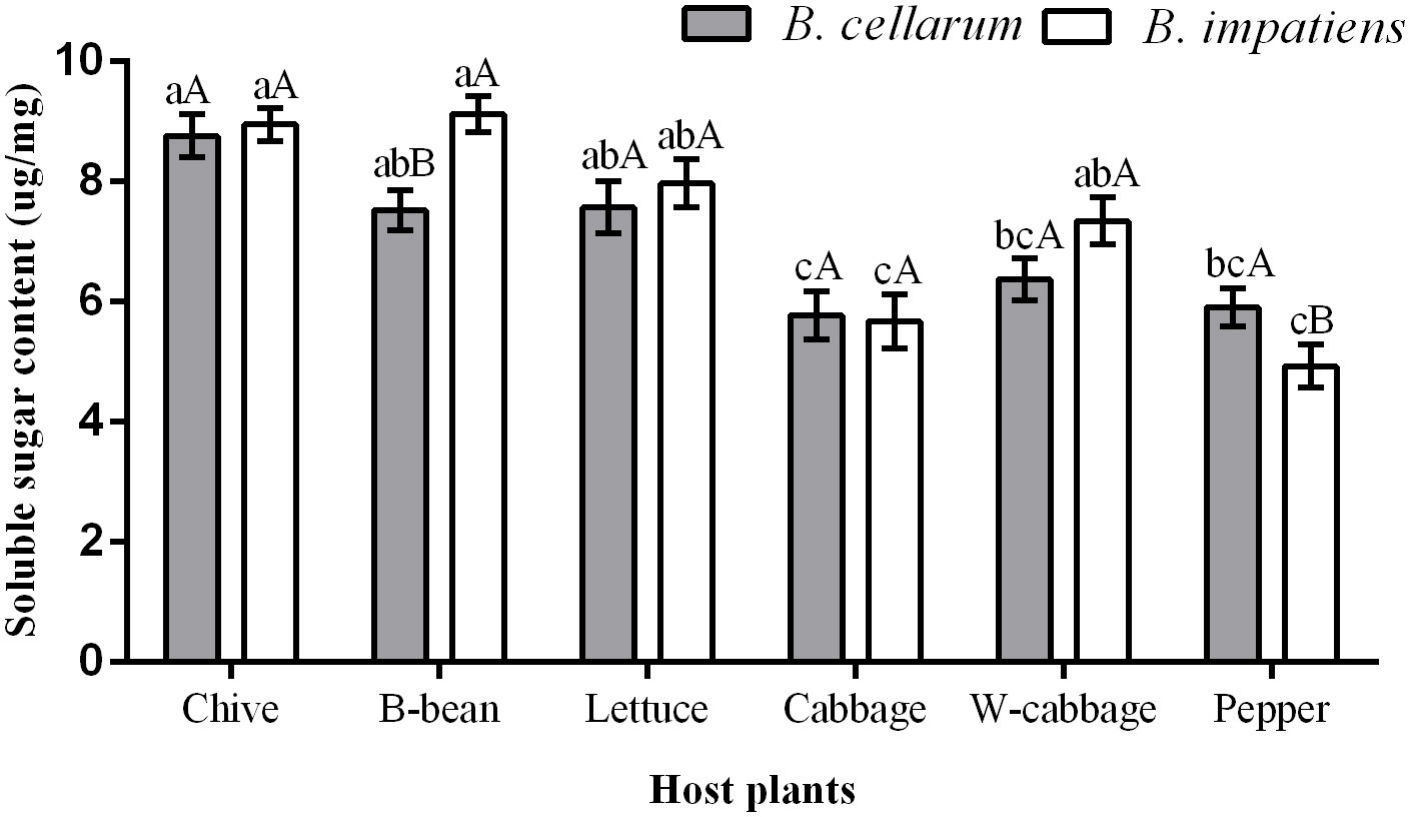
Soluble sugar content in *B*. *cellarum* and *B*. *impatiens*. Values are the means ± standard error. Different lowercase letters indicate significant differences between the two *Bradysia* species on different host plants by Tukey’s HSD (*P < 0*.*05*), while different uppercase letters represent significant differences between the two *Bradysia* species on the same host plant by Tukey’ s HSD (*P < 0*.*05*).

Soluble sugar content of *B*. *cellarum* and *B*. *impatiens* reared on the six host plants are showed in Fig 2. The highest sugar content was examined in *B*. *cellarum* when reared on chive, which was 8.68 mg/g, followed by 7.57 μg/mg, 7.53 μg/mg, and 6.38 μg/mg when reared on lettuce, B-bean and W-cabbage, respectively. Soluble sugar content was low when reared on pepper (5.91 μg/mg) and cabbage (5.78 μg/mg) as compared to those reared on other plants. However, the highest soluble sugar content of *B*. *impatiens* occurred on B-bean (9.13 μg/mg), followed by chive (8.95 μg/mg), lettuce (7.97 μg/mg), W-cabbage (7.34 μg/mg), and cabbage (5.68 μg/mg). Moreover, low levels of soluble sugar was detected in *B*. *impatiens* when reared on pepper (4.93 μg/mg). There were significant differences in the soluble sugar content between *B*. *cellarum* and *B*. *impatiens* when fed on B-bean and pepper (*P < 0*.*05*). We also found that *B*. *cellarum* and *B*. *impatiens* obtained high levels of soluble sugar when fed on chive, B-bean and lettuce compared those fed on cabbage, W-cabbage and pepper.

### Glycogen content in *B*. *cellarum* and *B*. *impatiens*

**Fig 3:**
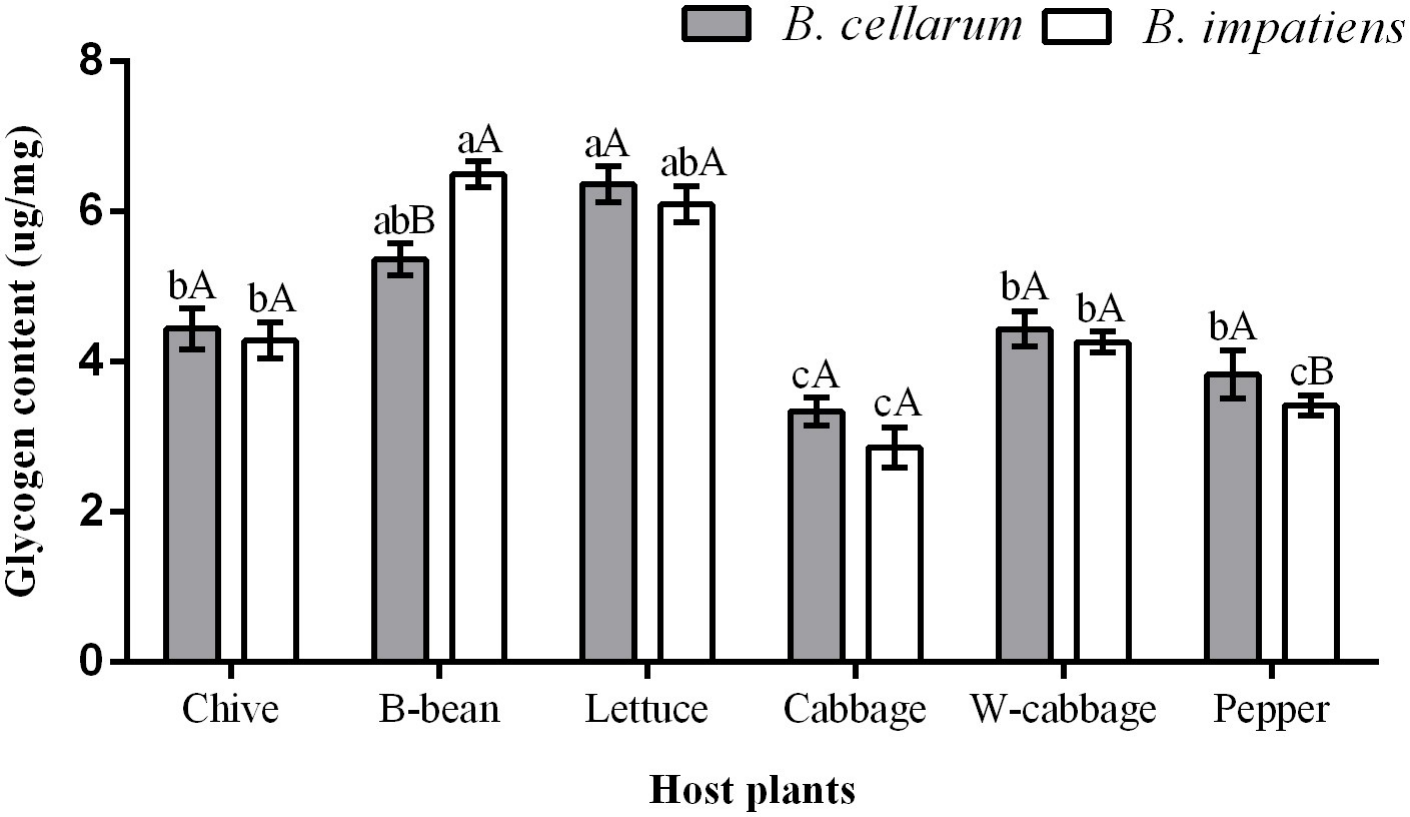
Glycogen content in *B*. *cellarum* and *B*. *impatiens*. Values are the means ± standard error. Different lowercase letters indicate significant differences between the two *Bradysia* species on different host plants by Tukey’s HSD (*P < 0*.*05*), while different uppercase letters represent significant differences between the two *Bradysia* species on the same host plant by Tukey’ s HSD (*P < 0*.*05*).

Glycogen content of *B*. *cellarum* and *B*. *impatiens* across the various host plants is presented in Fig 3. The highest levels were detected in *B*. *cellarun* when reared on lettuce (6.37 μg/mg), followed by B-bean (5.36 μg/mg), chive (4.44 μg/mg), W-cabbage (4.43 μg/mg), and pepper (3.83 μg/mg), while the lowest occurred on cabbage (3.34 μg/mg). Glycogen content of *B*. *impatiens* reared on the six host plants were 6.50 μg/mg on B-bean, 6.106 μg/mg on lettuce, 4.28 μg/mg on chive, 4.26 μg/mg on W-cabbage, 3.42 μg/mg on pepper, and 2.86 μg/mg on cabbage in turn from high levels to low levels. Meanwhile, similar levels in glycogen were also examined in *B*. *cellarum* when reared on the six host plants (Fig 3). Although significant differences (*P < 0*.*05*) were detected in glycogen content between *B*. *cellarum* and *B*. *impatiens* when fed on B-bean and pepper, either *B*. *cellarum* or *B*. *impatiens* obtained higher glycogen when fed on chive, B-bean, lettuce, and W-cabbage than on cabbage and pepper.

### Total fat content in *B*. *cellarum* and *B*. *impatiens*

**Fig 4:**
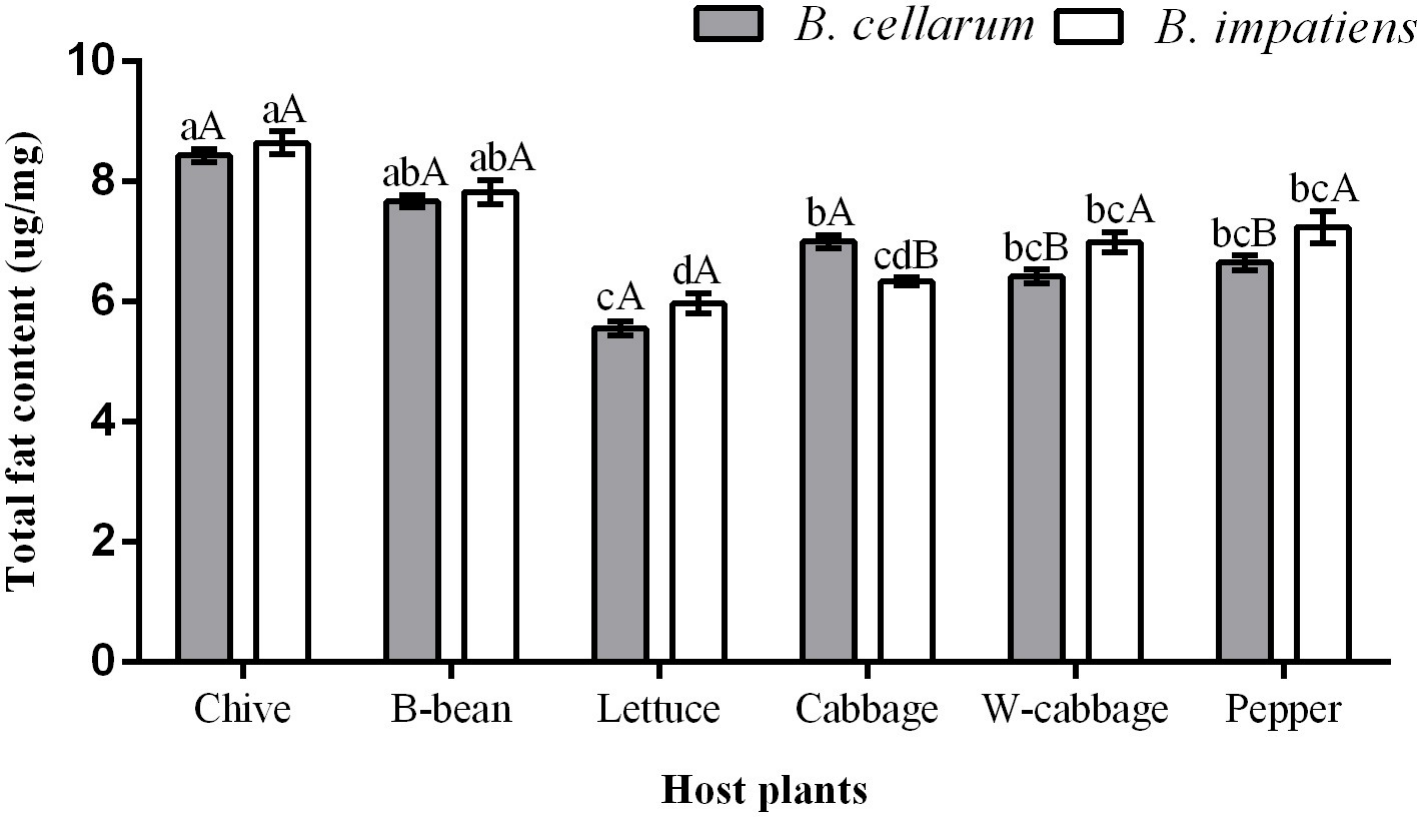
Total fat content in *B*. *cellarum* and *B*. *impatiens*. Values are the means ± standard error. Different lowercase letters indicate significant differences between the two *Bradysia* species on different host plants by Tukey’s HSD (*P < 0*.*05*), while different uppercase letters represent significant differences between the two *Bradysia* species on the same host plant by Tukey’ s HSD (*P < 0*.*05*).

The total fat content accumulated in *B*. *cellarum* and *B*. *impatiens* when reared on the six host plants are showed in Fig 4. The highest levels were examined in *B*. *cellarum* (8.43 μg/mg) when fed on chive. The total fat accumulated in *B*. *cellarum* when reared on B-bean, cabbage, pepper, and W-cabbage, which were 7.67, 7.00, 6.66 and 6.42 μg/mg, respectively. The lowest levels were detected in the insects (5.56 μg/mg) when fed on lettuce. The trends in the total fat contents in *B*. *impatiens* were similar to those in *B*. *cellarum* reared on the six host plants, although there are significant difference (*P < 0*.*05*) in the levels between the two species when reared on cabbage, W-cabbage and pepper.

### Neutral fat content in *B*. *cellarum* and *B*. *impatiens*

**Fig 5:**
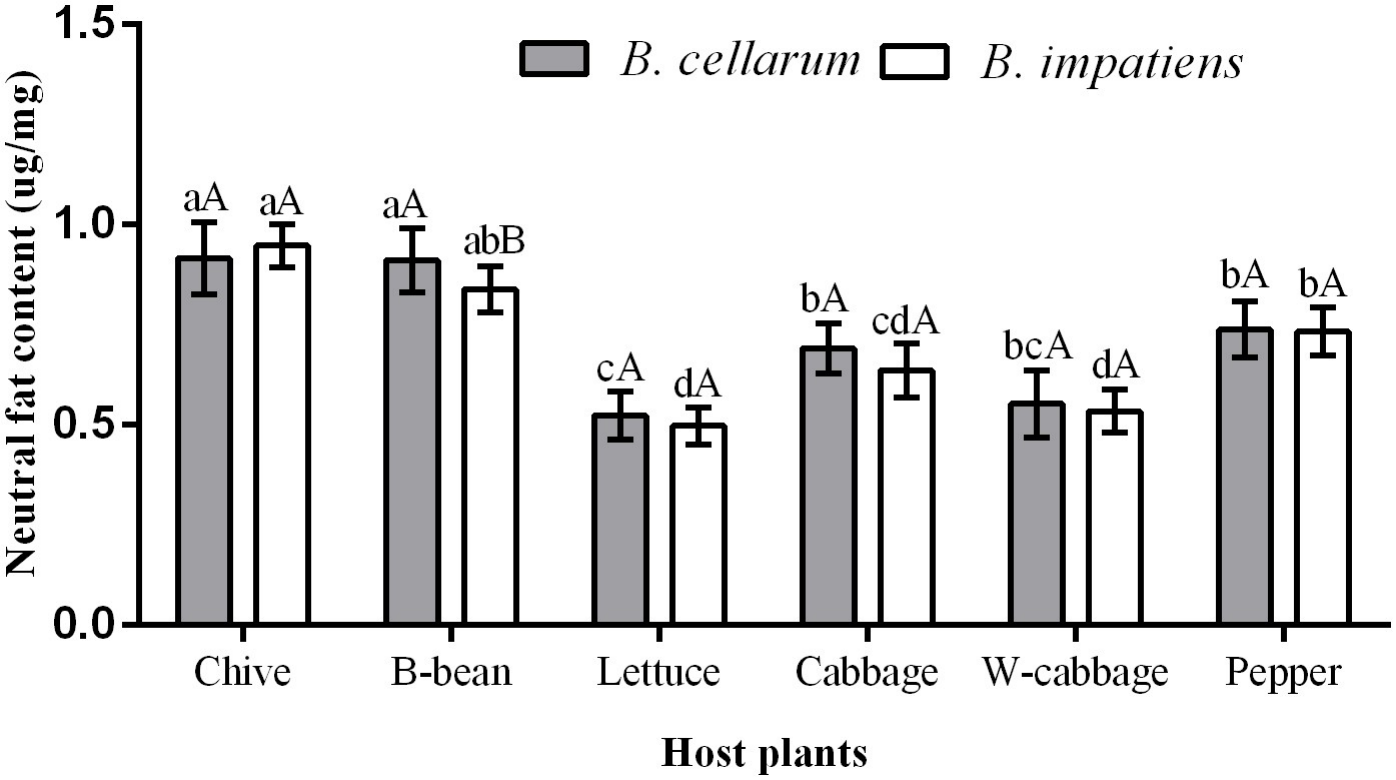
Neutral fat content in *B*. *cellarum* and *B*. *impatiens*. Values are the mean ± standard error. Different lowercase letters indicate significant differences between the two *Bradysia* species on different host plants by Tukey’s HSD (*P < 0*.*05*), while different uppercase letters represent significant differences between the two *Bradysia* species on the same host plant by Tukey’ s HSD (*P < 0*.*05*).

Feeding on six host plants exerted influences on the neutral fat content of *B*. *cellarum* and *B*. *impatiens* (Fig 5). The levels in *B*. *cellarum* reared on chive (0.92 μg/mg), and B-bean (0.91 μg/mg) were significantly higher than on pepper (0.74 μg/mg), and cabbage (0.69 μg/mg). *B*. *cellarum* accumulated low levels of the neutral fat when reared on lettuce (0.52 μg/mg), and W-cabbage (0.55 g/mg). Similar trends in the neutral fat levels were also examined in *B*. *impatiens* with feeding host plants, although there was a significant difference between the two insect species when reared on B-bean.

### Protein content in *B*. *cellarum* and *B*. *impatiens*

**Fig 6:**
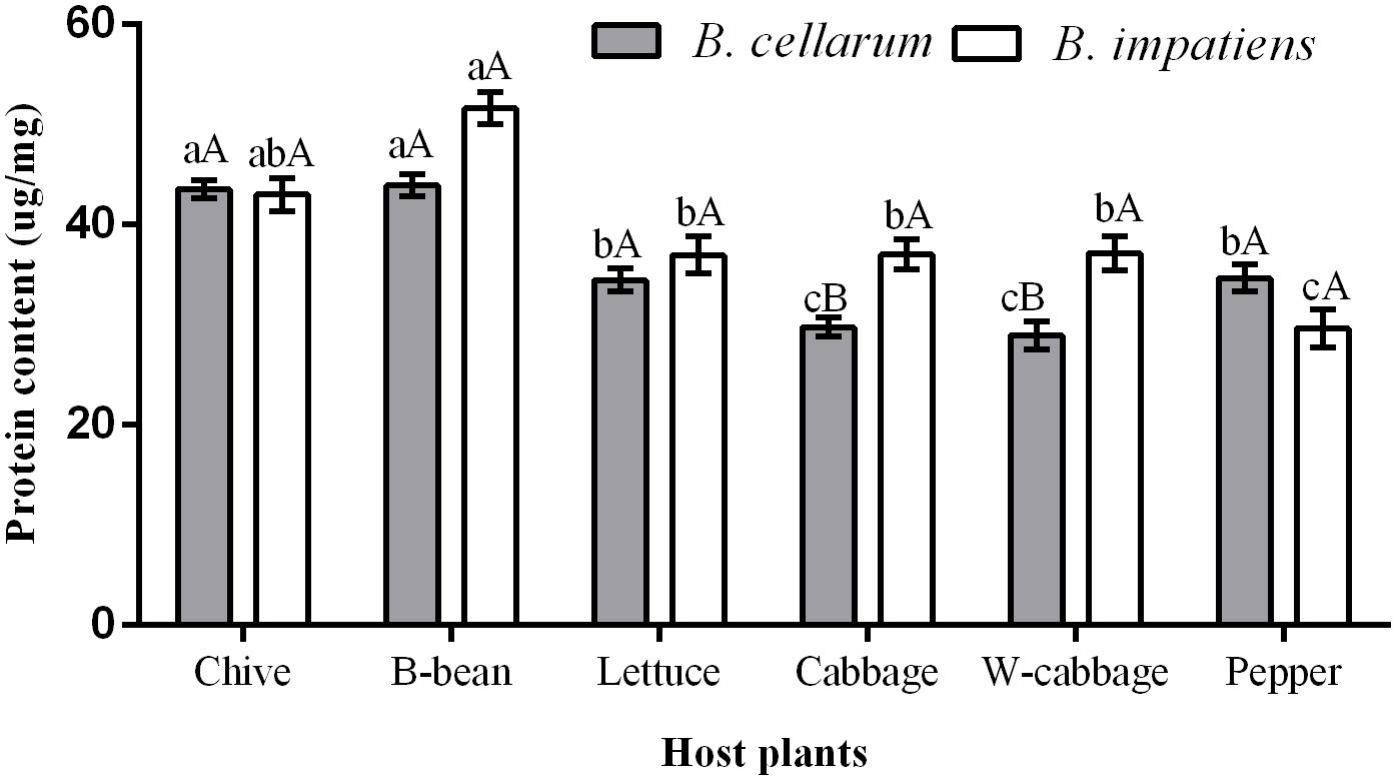
Protein content in *B*. *cellarum* and *B*. *impatiens*. Values are the mean ± standard error. Different lowercase letters indicate significant differences between the two *Bradysia* species on different host plants by Tukey’s HSD (*P < 0*.*05*), while different uppercase letters represent significant differences between the two *Bradysia* species on the same host plant by Tukey’ s HSD (*P < 0*.*05*).

The protein contents in *B*. *cellarum* when reared on B-bean (43.94 μg/mg) and chive (43.57 μg/mg) were significantly higher than those on pepper (34.67 μg/mg), lettuce (34.47 μg/mg), cabbage (29.79 μg/mg), and W-cabbage (28.99 μg/mg) (Fig 6). In *B*. *impatiens*, the protein levels fed on B-bean (51.61 μg/mg) were significantly higher than those on chive (43.02 μg/mg), W-cabbage (37.17 μg/mg), cabbage (37.02 μg/mg), lettuce (36.99 μg/mg), and pepper (29.69 μg/mg) (*P < 0*.*05* for all). Although the two insect species displayed similar trends in protein levels with feeding host plants, there was a significant difference in protein content between the *B*. *cellarum* and *B*. *impatiens* when reared on cabbage and W-cabbage.

### Correlation analysis of nutrients between host plants with those of *B*. *cellarum* and *B*. *impatiens*

Protein content in *B*. *cellarum* was significantly positively correlated with protein content (*P < 0*.*01*) and free amino acid content (*P < 0*.*05*) of the host plant roots, with correlation coefficients of 1.00 and 0.868, respectively (Table 1). Neutral fat content in *B*. *cellarum* had a significantly positive correlation with free amino acid content and the starch content of the host plant roots, with correlation coefficients of 0.840 and 0.823, respectively (*P < 0*.*05*; Table 1). The total fat content in *B*. *cellarum* was significantly correlated with the contents of soluble sugars and starch in host plants. Glycogen content in *B*. *cellarum* had a negative correlation with plant soluble sugar content. Starch content in the host plants was significantly correlated with the content of total fat and neutral fat in the insect.

**Table 1:** Correlation analysis of nutrients between host plant roots and *B*. *cellarum* and *B*. *impatiens*.

Correlation coefficient			Content of nutrients in host plant root (mg/g)			
			Protein	Free amino acid	Soluble sugar	Starch
Nutrients in body (μg/mg)	Protein	*B*. *cellarum*	1.000**	0.868*	0.489	0.808
	Total fat		0.701	0.750	0.865*	0.854*
	Neutral fat		0.840*	0.791	0.648	0.823*
	Soluble sugar		0.793	0.674	0.556	0.658
	Glycogen		0.341	0.242	-0.322	0.097
	Protein	*B*. *impatiens*	0.815*	0.863*	0.381	0.907*
	Total fat		0.839*	0.834*	0.844*	0.800
	Neutral fat		0.840*	0.764	0.735	0.784
	Soluble sugar		0.714	0.778	0.432	0.762
	Glycogen		0.514	0.528	-0.207	0.384

“*” indicates a significant correlation at the 0.05 level,

“**” indicates a significant correlation at the 0.01 level.

As for *B*. *impatiens*, all of the contents of protein, total fat and neutral fat were significantly positively correlated with the protein content of the host plant roots, with correlation coefficients of 0.815, 0.839 and 0.840, respectively (Table 1). The contents of protein and total fat in the *B*. *impatiens* was significantly (*P < 0*.*05*) positively correlated with the free amino acid content of host plant roots, with correlation coefficients of 0.863 and 0.834, respectively. The contents of protein and total fat in *B*. *impatiens* was significantly (*P < 0*.*05*) positively correlated with soluble sugar in host plant roots. Glycogen content in *B*. *impatiens* was negatively correlated with soluble sugar in host plant roots. There was a highly significant (*P < 0*.*01*) correlation between protein content of *B*. *impatiens* and the starch content in roots with a correlation coefficient of 0.907.

## Discussion

In this study, we found that B-bean and chive roots contained high levels of the protein, free amino acid, soluble sugar and starch as compared to the other host plants. Correspondingly, the two *Bradysia* species obtained much protein when they were reared on these two plants. Furthermore, the contents of protein and free amino acid in plant roots were significantly correlated with protein content in the two *Bradysia* species. It is reported that *B*. *cellarum* and *B*. *impatiens* have more robust adaptability with short developmental duration and high oviposition, net reproductive rate and intrinsic rate value when they were fed on chive and B-bean [11]. These parameters might be due to high level of protein and free amino acid contained these host plants, which promote the growth, development and fecundity of the *Bradysia* species. The western flower thrip *Frankliniella occidentalis* moves to pollen from leaves once the plants bloom because the pollen contains higher levels of protein than leaves [34]. Therefore, we presumed that the two *Bradysia* species prefer a host plant with high levels of protein and free amino acid contents.

Several studies demonstrate that the significant contributions of amino acid and other nutrients in some insect species [35]. For instance, high soluble sugar level improved survival, development stage and completion of larval development in *Exeristes roborator* [35]. Thompson reported similar effects of amino acid level on larval development, for ectoparasitoid wasp [36]. In the present study, it was found that the high host plants nutrient correlated positively with the nutrient content in the insets. This is an indication that, host plant nutritional quality plays vital role in the nutrition quality in insects. Nutrition quality in insect is reported to support limited development and supply sufficient quantity to ensure development of one generation [26]. Moreover, it can contribute significantly to insect’s spermatogenesis (the manufacture of sperms), oogenesis (the manufacture of eggs), reproduction, detoxification process and resistance mechanism as reported by previous studies [26]. This, accordingly, can improve insets survival, development stage and successful completion of larval development. We therefore speculate that, nutrition quality of host plants and insect interaction should be well examined.

Our results revealed that soluble sugar content was significantly higher in chive, W-cabbage and B-bean than lettuce, cabbage and pepper. Moreover, we also found that soluble sugar content in the body of the two *Bradysia* species, and especially *B*. *impatiens*, accumulated high levels of soluble sugar when reared on chive and B-bean, which seemed to be consistent with the report that *B*. *impatiens* heavily attacked chive and B-bean [13]. Other studies reveal that soluble sugar content in *Brassica campestris* L. ssp. *chinensis var*. *utilis* Tsen et is negatively correlated with aphid resistance, owing to the amount of soluble sugar that positively promote the fecundity of *Spodptera exigua* [21, 37, 38]. A recent study report that *B*. *cellarum* reared on chive have significantly long female and male longevity, as well as high oviposition and survival rate [39]. However, our results showed that the soluble sugars might contribute to total fat content in the *Bradysia* species to a great extent, which was consistent with the report that carbohydrate provides energy for insect growth and flight [21]. In addition, soluble sugar content in the host plant roots was negatively correlated with glycogen content in *Bradysia* species. Glycogen is the energy storage material of insects, known as animal starch, which is used as the energy storage material for embryo development, and is correlated with the reproduction of insects [40]. It is also the essential animal version of starch and the main energy storage material in organisms [40]. There might be a tradeoff between the growing development and reproduction of the two *Bradysia* species, as the adults do not feed.

The larvae of *B*. *cellarum* reared on chive had much higher soluble sugar content than those reared on B-bean. However, the larvae of *B*. *impatiens* reared on B-bean had much higher soluble sugar content than those reared on chive. In addition, *B*. *cellarum* obtained a high level of glycogen when they were reared on lettuce, while *B*. *impatiens* obtained it when reared on B-bean. These physiological differences between the two *Bradysia* species need to be further investigated.

In summary, the nutrient content in an insect body is an important guarantee for their growth, development, and reproduction. Our study demonstrated that the host plant nutrients, especially protein, free amino acid, and soluble sugar, might strongly affect the nutrient contents of *B*. *cellarum* and *B*. *impatiens*. Correspondingly, we found that the chive and B-bean contained high contents of protein, free amino acid and soluble sugar. Hence, we proposed that rotation of those two plants should be avoided in the field to control for rapid population expansion and the damage caused by *B*. *cellarum* and *B*. *impatiens*. Such a strategy could cause a decrease in the continuous supply of their nutrient requirements from these host plants, which could further weaken their behaviour and performance.

## Supporting information

S1 File The study’s minimal data set. (XLS)
